# Dihydromyricetin ameliorated nonalcoholic steatohepatitis in mice by regulating the composition of serous lipids, bile acids and ileal microflora

**DOI:** 10.1186/s12944-023-01871-7

**Published:** 2023-08-02

**Authors:** Xiaolei Miao, Ping Luo, Jiao Liu, Junjun Wang, Yong Chen

**Affiliations:** 1 Hubei Province Key Laboratory of Biotechnology of Chinese Traditional Medicine, National & Local Joint Engineering Research Center of High-throughput Drug Screening Technology, State Key Laboratory of Biocatalysis and Enzyme Engineering, Hubei University, Wuhan, 430062 China; 2 School of Pharmacy, Xianning Medical College, Hubei University of Science and Technology, Xianning, 437100 China

**Keywords:** Dihydromyricetin, Nonalcoholic steatohepatitis, Lipidomic, Bile acids, Gut microbiota

## Abstract

**Background:**

Dihydromyricetin (DMY) is a natural flavonoid with anti-nonalcoholic steatohepatitis (NASH) activity. However, the effects of DMY on the composition of lipids and bile acids (BAs) in serum, and gut microbiota (GM) in ileum of mice with NASH are not clear.

**Methods:**

After male C57BL/6 mice was fed with methionine and choline deficiency (MCD) diet and simultaneously administered with DMY (300 mg/kg/day) by gavage for 8 weeks, the pathological changes of liver tissue were observed by Oil Red O, hematoxylin eosin and Masson staining, the levels of serum alaninea minotransferase, aspartate aminotransferase and liver triglyceride, malonic dialdehyde were detected by the detection kits, the composition and contents of serum lipids and BAs were detected by Liquid Chromatograph-Mass Spectrometry, the mRNA levels of hepatic BAs homeostasis-related genes were detected by RT-qPCR, and microbiological diversity in ileum was analyzed by 16S rDNA sequencing.

**Results:**

The results showed that the significant changes including 29 lipids, 4 BAs (23-nor-deoxycholic acid, ursodeoxycholic acid, 7-ketodeoxycholic acid and cholic acid), 2 BA transporters (*Mrp2* and *Oatp1b2*) and 8 GMs between MCD and DMY groups. Among them, DMY treatment significantly down-regulated 21 lipids, 4 BAs mentioned above, the ratio of *Firmicutes/Bacteroidota* and the abundance of *Erysipelotrichaceae*, *Faecalibacuium,* significantly up-regulated 8 lipids and 5 GMs (*Verrucomicrobiota*, *Bacteroidota, Actinobacteria*, *Akkermansiaceae* and *Akkermansia*).

**Conclusions:**

The results suggested that DMY may alleviate MCD diet-induced NASH through decreasing the serum levels of toxic BAs which regulated by liver *Oatp1b2* and *Mrp2*, regulating the metabolism of related lipids, and up-regulating intestinal probiotics (*Actinobacteria* and *Verrucomicrobiota* at the phylum level; *Akkermansiaceae* at the family level; *Akkermansia*at at the genus level) and inhibiting intestinal harmful bacteria (*Firmicutes* at the phylum level; *Erysipelotrichaceae* at the family level; *Faecalibaculum* at the genus level).

**Supplementary Information:**

The online version contains supplementary material available at 10.1186/s12944-023-01871-7.

## Introduction

Non-alcoholic steatohepatitis (NASH) is a common chronic liver disease, characterized by bullae steatosis, hepatocellular balloonlike degeneration, lobular inflammation and varying degrees of fibrosis [1–3]. Abnormal lipids accumulation especially triglyceride (TG) in hepatocytes is considered to be the basis of the formation and development of NASH [4, 5].

It has been reported that bile acids (BAs)—gut microbiota (GM) axis plays an important role in the pathogenesis of NAFLD/NASH [6]. BAs are the final products of cholesterol metabolism mainly by hepatic cells. According to its source, it can be divided into the primary BAs (i.e., cholic acid [CA] and chenodeoxy-CA [CDCA]) generated by liver cells and the secondary BAs (i.e., deoxy-CA [DCA] and lithocholic acid [LCA]) formed via de-conjugation and de-hydroxylation by the resident bacteria of the distal small intestine and colon. BAs mainly exist in the enterohepatic circulation system and play various physiological functions through recycling, such as regulating cholesterol clearance, GM composition and hepatic glucolipid metabolism [7, 8]. Under normal physiological condition, the level of BAs in the body remains stable. When BAs homeostasis is destroyed, oxidative stress and inflammation can be activated, leading to cholestasis, hepatic steatosis and fibrosis [9]. The serum BAs level in NASH patients were significantly increased [10], and the severity of the disease was positively correlated with BAs synthesis and its serum level [11]. Additionally, GM can regulate the metabolism of endogenous ethanol, choline and BAs by influencing farnesoid X receptor (FXR) signal transduction [12, 13]. Human GM mediates the occurrence and progression of NAFLD/NASH through its metabolites, such as BAs, amino acids and short-chain fatty acids [6, 14]. There is evidence of changes in the content and composition of GM in the small intestine of NASH patients [15], and such changes can affect the body's energy homeostasis, leading to liver steatosis [16], as well as changes in intestinal permeability and metabolic endotoxemia associated with liver inflammation and fibrosis [17, 18]. Therefore, the enteric-liver axis is considered as a new target for prevention and treatment of NASH.

Dihydromyricetin (DMY) is a natural type of flavonoids existed in *Ampelopsis grossedentata (Hand.-Mazz.)* and traditionally used to treat fever or cough [19]. As far as we know, in addition to anti-inflammatory, antioxidant and hepatoprotective pharmacological activities [19], DMY also has significant anti-NASH effects. In brief,

DMY promoted AMPK by inhibiting the expression of PPAR and phosphorylation of serine/threonine kinase Akt, and improved lipids deposition induced by oleic acid in L02 and HepG2 cells [20]. DMY reduced the levels of serum ALT, AST, TC, TG, LDLc and nonestesterified fatty acid, enhanced the synthesis and transport of intrahepatic BAs, and inhibited the reabsorption of ileal BAs in ob/ob mice by regulating FXR signaling pathway [21]. DMY inhibited the de novo lipid synthesis of fat in the liver of obese mice through FXR-SREBP-1C pathway, improved mitochondrial respiration capacity and redox homeostasis by regulating SIRT3 signaling, reduced liver lipid deposition of high fat diet (HFD) fed mice and palmic acid-treated mouse primary hepatocytes [22]. Moreover, DMY improved glucolipid metabolism and inflammation of NAFLD patients [23]. However, the effects of DMY on ileum GM and serum lipids and BAs of NASH mice are unclear.

Considering the important role of the enteric-liver axis in BA and lipid metabolism and its close relationship with NASH, mice fed by methionine and choline deficiency (MCD) diet were used in this work to study DMY-induced effects on the composition of serum lipids and BAs detected by LC–MS, as well as the diversity of ileal GM assayed by 16S rDNA sequencing. The results have certain reference value for elucidating the mechanism of DMY in preventing NASH.

## Materials and methods

### Chemicals and reagents

DMY and obeticholic acid (OCA) (purity ≥ 98%) were got from Shanghai Source Leaf Biological Technology Co., Ltd (Shanghai, China). MCD and MCS (choline and methionine sufficient) diet were from Nantong Trophy Feed Technology Co., Ltd. (Jiangsu, China). Lipid and 50 BA standards were purchased from CNW/IsoReag (Duesseldorf, Germany). RNase A was purchased from Promega. Trizol was from Invitrogen (California, USA). FSQ-301 ReverTra Ace® qPCR RT Kit was from Toyobo Co., Ltd (Osaka, Japan). SYBR Green I fluorescent RT PCR kit was from Bio-Rad Laboratories (California, American). Phusion® High-Fidelity PCR Master Mix was purchased from NEB (Inc., MA, USA). TruSeq® DNA PCR-free sample preparation kit was purchased from Illumina (California, USA). Qiagen Gel Extraction Kit was purchased from Qiagen (Duesseldorf, Germany). The detection kits of ALT, AST, TG and MDA were from Nanjing Jiancheng Bioengineering Research Institute (Jiangsu, China).

### Animals and treatment

**Fig. 1:**
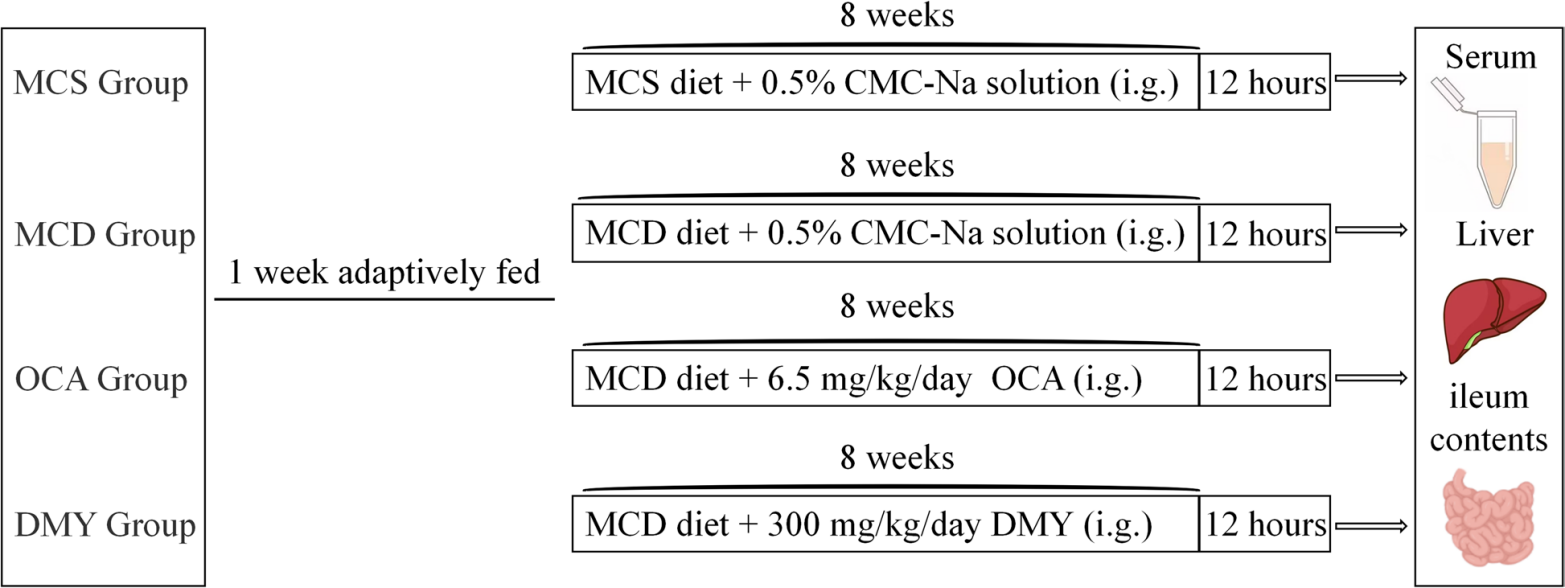
The flowchart of animal modeling and drug administration treatment

The 6–8 weeks SPF male C57BL/6 mice were from Sanxia University (No. SCXK (E) 2022–0012). Mice were maintained in an SPF animal house with 12 h cycles of light and darkness. The room temperature is maintained at 22 ± 2°C and the humidity is 50–60%. All animal experiments were conducted in the SPF laboratory animal room at Hubei University (No. SYXK (E) 2022–0134). Mice were randomly divided into four groups, and subjected to the following treatments: MCS group, MCD group, DMY group and positive control (OCA) group, 7 mice in each group. Mice in MCS group and other groups were given respectively with MCS and MCD diet for 8 weeks, and mice in DMY and OCA groups were given the corresponding drug by gavage once a day during the MCD diet feeding period. The dosage of DMY was 300 mg/kg/day [22], and OCA was 6.5 mg/kg/day converted from the clinically recommended dose for adults [24]. Mice in MCS and MCD groups administrated with an equal amount of 0.5% CMC-Na solution. Mice were monitored daily for their general health and their body weight once a week. At the end of 8 weeks administration, blood samples were collected from orbital venous plexus. Liver and ileum contents of each mouse were collected after sacrifice by cervical dislocation for subsequent experiments. The specific experimental schematic is shown in Fig. 1.

### Biochemical parameters analysis

The activity of serum ALT and AST, and hepatic TG and MDA contents were determined following the instructions of test kits.

In brief, the blood samples were centrifuged at 3500 rpm for 10 min at 4°C to obtain serum. The activity of AST/ALT was calculated by measuring the absorbance of the product produced by the reaction of 2,4-dinitrophenylhydrazine hydrochloride with pyruvic acid.

The liver tissue was excised, homogenized with 10 volumes of ice-cold normal saline, and the supernatant was separated at 3500 rpm for 10 min. The 10% liver tissue homogenate was added to TG working solution. The absorbance of the red quinone formed by the reaction of hydrogen peroxide with 4-aminoantipyrine and p-chlorophenol was measured. The corresponding protein concentration was also measured. The TG content was calculated according to the instructions. For MDA detection, 10% tissue homogenate was added to MDA working solution. The absorbance of the red condensation product formed by the reaction of malondialdehyde with thiobarbituric acid and protein concentration were measured. The MDA content was calculated according to the instructions.

### Liver histopathologic analysis

The liver tissue was quickly fixed in 4% paranormal-dehyde for 24 h and made into 4 µm paraffin sections for H&E and Masson stainning. Liver tissue was frozen and cut into 10 μm frozen section for Oil Red O staining.

### Serum untargeted lipidomic investigation

Serum samples were pre-treated according to the method described in literature [25]. In short, 50 μL serum sample, 1 mL mixture containing methanol, MTBE and internal standard mixture and 200 μL water were mixed evenly. After centrifugation (12000 rpm, 10 min), 200 μL supernatant was collected and dried. The extract was redissolved in 200 μL mobile phase.

UPLC coupled with QTRAP mass spectrometer 6500 + (SCIEX, USA) was used for lipidomic analysis. Each sample was separated by C_30_ column (2.6 µm, 2.1 mm × 100 mm). The mobile phase A was 60% acetonitrile and 40% water (containing 0.1% CH_2_O_2_ and 10 mmol/L CH_5_NO_2_). The mobile phase B was 10% acetonitrile and 90% isopropanol (containing 0.1% CH_2_O_2_ and 10 mmol/L CH_5_NO_2_). The flow rate was 0.35 mL/min. The elution gradient was listed in Table 1. The mass spectrometry analysis was using an electrospray ionization ESI ^+^/ESI^−^ mode.

**Table 1:** The gradient elution of lipids and BAs detection

	Time (min)	Pump B concentration
Lipids detection	0–2	20–30%
	2–4	30–60%
	4–9	60–85%
	9–14	85–90%
	14–15.5	90–95%
	15.5–17.3	95%-20%
	17.3–20	20%
BAs detection	0–0.5	5%-40%
	0.5–4.5	40–50%
	4.5–7.5	50–75%
	7.5–10	75%-95%
	10–12	95%-5%

After processing the raw data, it was imported to R software for multiple statistical analysis, including PCA and OPLS-DA. Value of variable importance (VIP) ≥ 1 and fold change (FC) of lipid content (≥ 2 or ≤ 0.5) were used as criteria to screen differential lipids. The metabolic pathway analysis was carried out by the KEGG database.

### Serum BAs analysis

50 μL serum sample, 200 μL methanol and 10 μL internal standard were mixed. And put the samples at -20°C for 10 min. Then centrifugate at 12000 rpm for 10 min. 200 μL supernatant was collected. The extract was evaporated to dryness, reconstituted in 100 μL mobile phase.

The BAs contents in serum were measured by ExionLC AD UPLC coupled with QTRAP® 6500 + Quadrupoletriple mass spectrometry from SCIEX Corp (SCIEX, USA). Chromatographic separations were performed with a C_18_ column (1.8 µm, 2.1 mm × 100 mm; Waters, USA). The mobile phase A was ultra-pure water (containing 0.01% CH_3_COOH and 5 mmol/L CH_3_COONH_4_). The mobile phase B was acetonitrile (containing 0.01% CH_3_COOH). The flow rate was 0.35 mL/min. The gradient elution was listed in Table 1. ESI^−^ and scheduled multiple reaction monitoring were adopted for mass spectrometry detection.

### Gut microbiota analysis

Total genomic DNA was extracted from the ileal contents and and tested for quality. Then PCR amplification was performed. The specific primers with Barcode (515F and 806R) were selected according to the sequencing region to be used. PCR products were tested with 2% agarose gel and purified. The library was constructed with the kit and sequenced on Illumina MiSeq 2 × 300. Uparse software was used for clustering, and sequences with 97% similarity were clustered into OTUs. Qiime software (Version 1.9.1) was used for α and β diversity analysis.

### RNA extraction and quantitative real-time PCR

Total RNA was extracted from the liver tissue (50–100 mg) of each mouse, and the quality and concentration of RNA was detected by nucleic acid dye on 1% agarose gel and Nucleic Acid Analyzer. Reverse transcription for cDNA synthesis with Reverse Transcription Kit. Then set the reaction conditions (Table S1) to complete the RT-qPCR reaction. The expression of target genes were calculated according to the instructions. The primer sequence was listed in Table S1.

### Statistical analysis

Experimental results were expressed as mean ± standard deviation. Multiple comparison among groups was carried out by Tukey's multiple range test in ANOVA analysis using SPSS 25.0 software. *P* value less than 0.05 indicated a significant difference and had statistical significance. Spearman method was used to analyze the relevance between GM and serum lipids and BAs.

## Result

### Effects of DMY on body weight and liver index

**Fig. 2:**
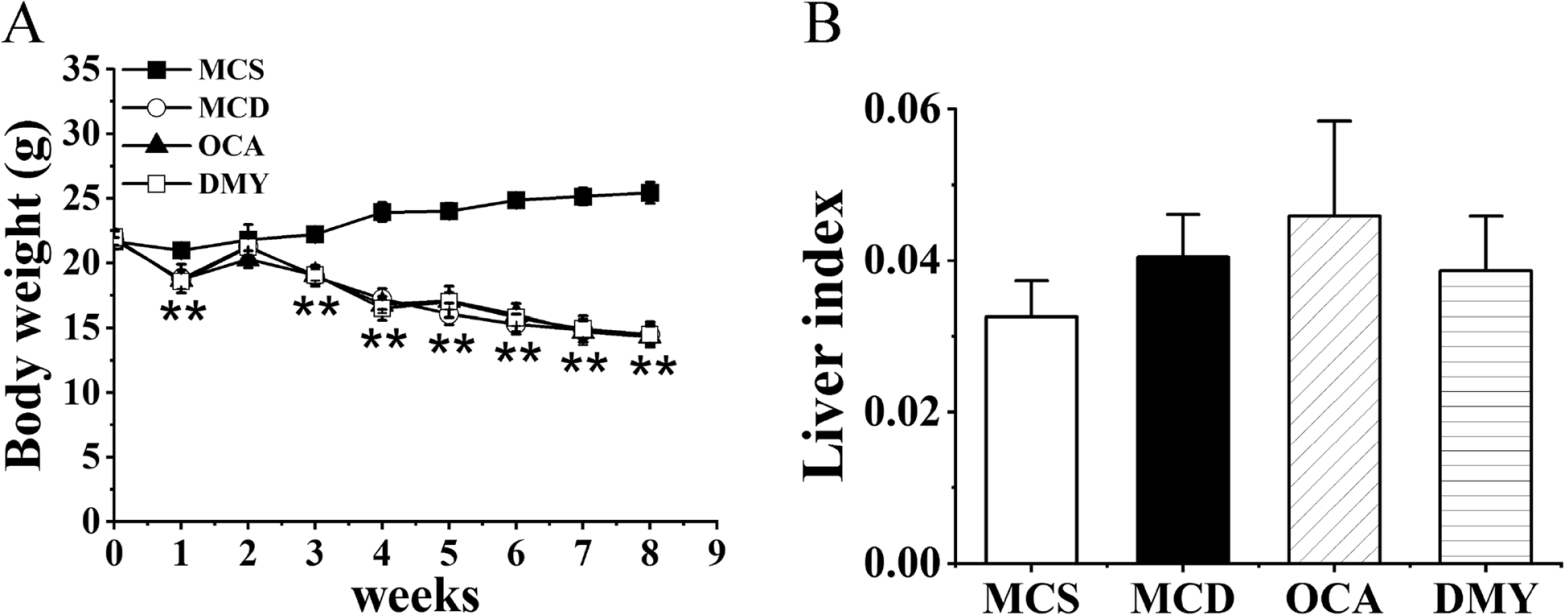
Body weight (**A**) and liver index (**B**) of the tested mice (*n* = 7). ** indicates comparison between MCS and MCD group and *P* < 0.01

Figure 2A showed the weight of the mice. Compared with the MCS group, the weight of mice in the MCD, DMY and OCA groups decreased significantly. Liver indexes were similar in all groups (Fig. 2B).

### Effects of DMY on biochemistry of liver and serum

**Fig. 3:**
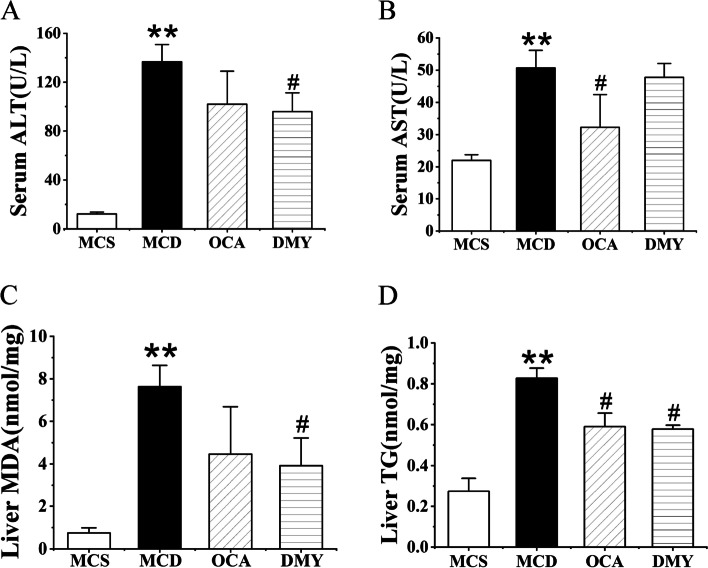
The contents of ALT (**A**) and AST (**B**) in serum, MDA (**C**) and TG (**D**) in liver of the tested mice (*n* = 7). ** indicates comparison with MCS group and *P* < 0.01. # indicates comparison with MCD group and *P* < 0.05

Compared with MCS group, the contents of serum ALT, AST and liver TG and MDA in mice fed MCD diet were significantly increased, indicating that the model was successfully established. After 8 weeks of DMY treatment, the contents of ALT, TG and MDA were significantly reduced. Meanwhile, OCA treatment also significantly reduced AST and TG contents (Fig. 3).

### Effects of DMY on liver pathomorphology

**Fig. 4:**
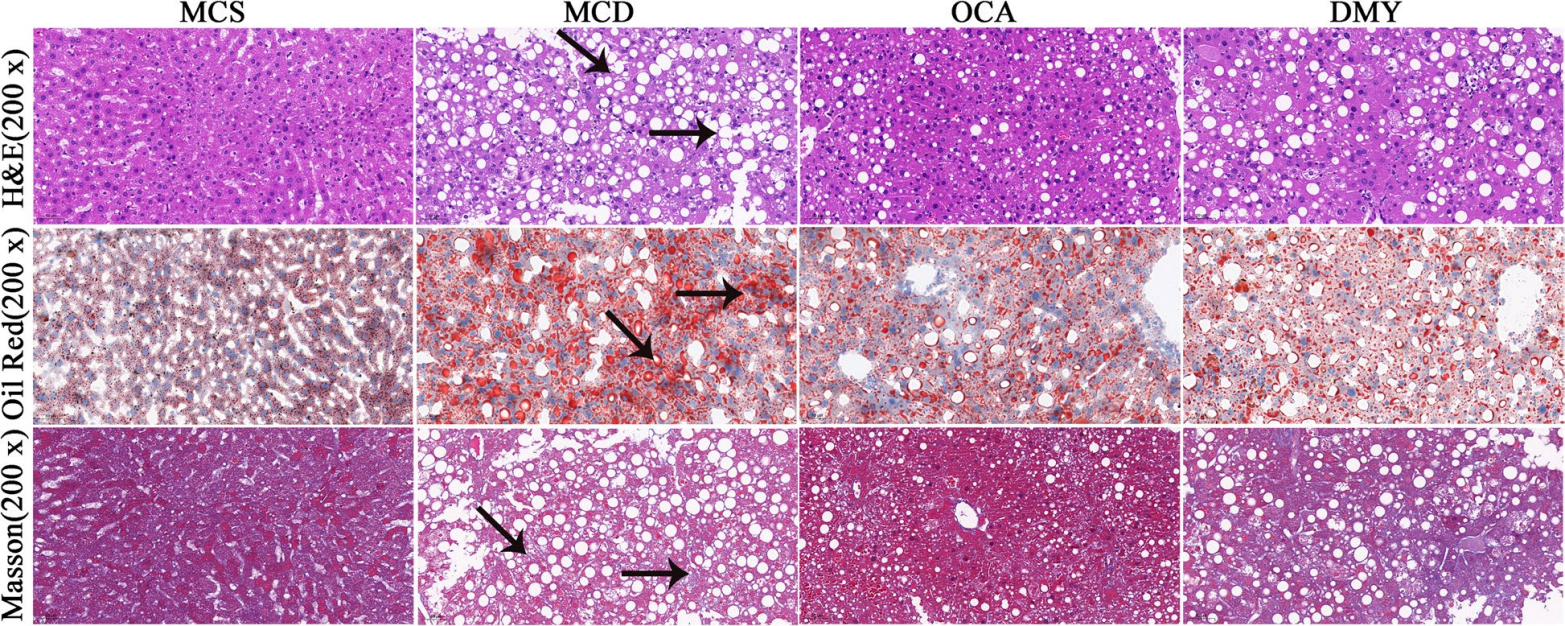
Pathological changes of liver in the tested mice evaluated by HE, Red Oil O and Masson staining (*n* = 7). Black arrows in HE staining indicate bigger cytoplasmic vacuolation, inflammation and hepatocellular ballooning respectively, in Red Oil O staining indicate bigger red lipid droplets, and in Masson staining indicate hepatic fibrosis

The results of pathological sections were shown in the Fig. 4. Compared with MCS group, the liver of MCD mice has many fat vacuoles and fat droplets, and swelling and balloon-like degeneration occurred, accompanied by inflammatory cell infiltration and fibrosis. DMY or OCA treatment significantly reduced the hepatic fat vacuoles, lipid droplets and hepatocyte ballooning, along with improved hepatic steatosis and lobular inflammation, whereas no improvement on fibrosis was observed. These results suggested that both DMY and OCA alleviated steatohepatitis in NASH mice, but OCA was slightly better than DMY in reducing the number of fat vacuoles.

### Effects of DMY on serum lipid profile

**Fig. 5:**
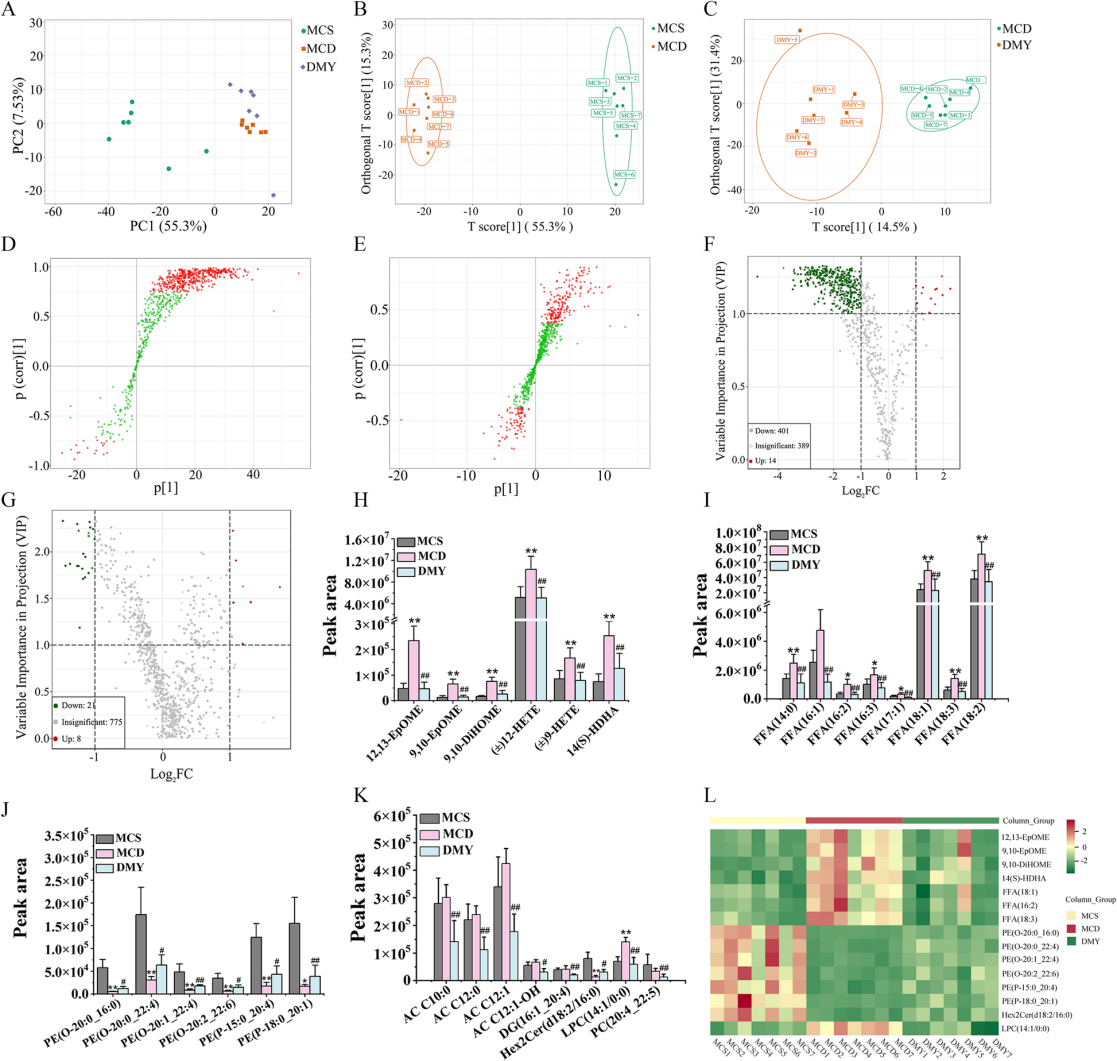
Model analysis of serum lipid metabolic profile in mice (*n* = 7). **A** PCA score plot. **B** OPLS-DA score plot between MCS group and MCD group. **C** OPLS-DA score plot between MCD group and DMY group. **D** S-shaped scatter plot between MCS group and MCD group. **E** S-shaped scatter plot between MCD group and DMY group. **F** Volcano plots of lipids between MCS and MCD groups. **G** Volcano plots of lipids between MCD and DMY groups. Intensities of representative differential eicosanoids (**H**), FFAs (**I**), PEs (**J**) and other differential lipids (**K**). **L** Heat map of 15 differential lipids among MCS, MCD and DMY groups. * or ** indicates comparison with MCS group and *P* < 0.05 or 0.01. # or ## indicates comparison with MCD group and *P* < 0.05 or 0.01

Lipidomics was used to analyze the differences of serum lipid composition among the mice in MCS, MCD and DMY groups. It can be seen from the PCA score map (Fig. 5A), MCS and MCD groups could be separated, DMY group was also separated from MCD group, and was closer to MCS group, suggesting that DMY treatment improved MCD-induced disorder of lipid metabolism.

Furthermore, the supervised OPLS-DA model was established to further identify differential lipid species. The OPLS-DA score map indicated that the MCS group was significantly separated from the MCD group (Fig. 5B), although MCD group and DMY group could also be distinguished (Fig. 5C), but the difference was not as significant as that between MCD group and MCS group. In the S-plots (Fig. 5D, E), Red dots indicate the lipids with VIP > 1, the green dots indicate the lipids with VIP < 1. As shown in Fig. 5F, 451 lipid species in MCD group were significantly changed in contrast to MCS group, including 23 aliphatic acyls (FA), 282 glycerophospholipids (GP), 44 sphingolipids (SP), 14 sterol lipids (ST) and 52 glycerides (GL). Among them, 401 lipids species were down-regulated (shaded green), 14 lipid species were up-regulated (shaded red). Compared with MCD group, there were 21 down-regulated lipid species and 8 up-regulated lipid species in DMY group (Fig. 5G-K), and 15 of 29 lipid species were the mutual differential lipid species among the three groups, including 7 up-regulated lipids (PE(O-20:0_22:4), (PE(O-20:0_16:0), PE(O-20:1_22:4), PE(P-18:0_20:1), PE(O-20:2_22:6), PE(P-15:0_20:4), Hex2Cer(d18:2/16:0)) and 8 down-regulated lipids (12,13-EpOME, FFA(18:3), 14(S)-HDHA, 9,10-EpOME, 9,10-DiHOME, FFA(18:1), LPC(14:1/0:0), FFA(16:2)) (Fig. 5L). Of note, the levels of the above 4 eicosanoids and 3 FFAs, as well as PE(O-20:1_22:4) and LPC(14:1/0:0) in DMY group, were almost consistent with those of MCS group.

**Fig. 6:**
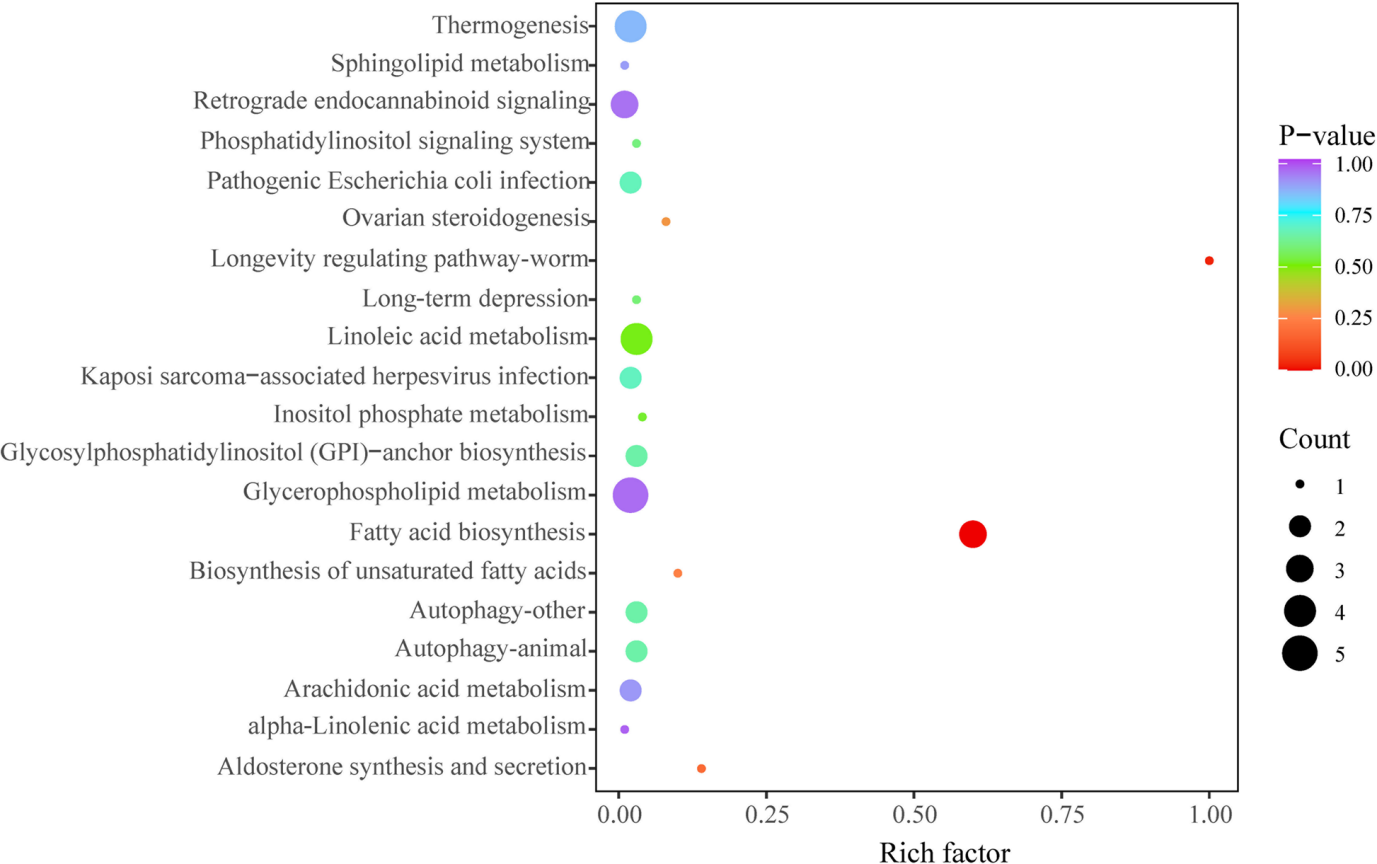
KEGG analysis of differential lipids enriched in pathways between DMY group and MCD group

Pathway enrichment analysis showed that 15 lipid species mentioned above was involved in 20 metabolic pathways (Fig. 6). Based on the *P*-value, the biosynthetic pathway of fatty acids is considered to be the main pathway involved in the regulation of lipid metabolism disorders in MCD mice by DMY.

### Effects of DMY on the composition of GM in ileum

The composition of GM in ileum was analyzed by using 16S rDNA sequencing. The statistical analysis of the α diversity indexes of different samples under the 97% consistency threshold was presented in Table 2. The Chao1, ACE and Shannon indexes of MCD group were lower than those of MCS group, but without significant difference. DMY treatment significantly increased these indexes as compared to MCD group, which suggested that DMY changed the α diversity of GM in ileum of MCD mice.

**Table 2:** The α diversity indexes of tested mice (*n* = 7)

Group	Chao1	ACE	Shannon
MCS	218.06 ± 12.58	226.54 ± 13.14	2.83 ± 0.20
MCD	192.31 ± 16.53	198.02 ± 13.73	2.50 ± 0.11
DMY	350.17 ± 32.37^##^	354.69 ± 34.12^##^	3.45 ± 0.33^##^

^##^ indicates comparison with MCD group and a *P* < 0.01

**Fig. 7:**
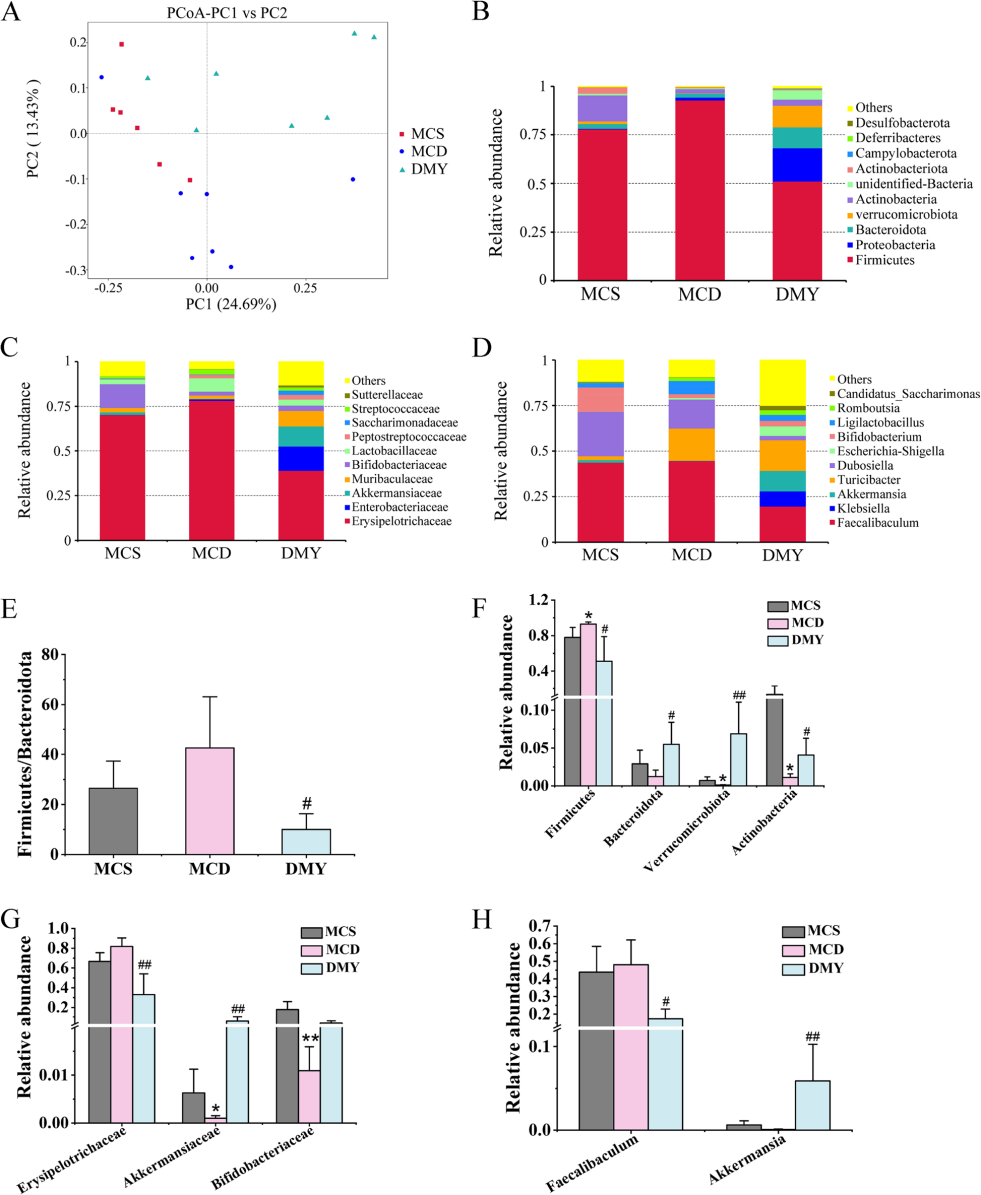
The composition of GM in the mice (*n* = 7). **A** PCoA of the GM; **B**-**D** Top 10 most abundant taxa (phylum, family and level genus, respectively). **E** Abundance proportion of Firmicutes and Bacteroidota. **F**–**H** Significantly altered GM (phylum, family and genus level, respectively). * or ** indicates comparison with MCS group and *P* < 0.05 or 0.01. # or ## indicates comparison with MCD group and *P* < 0.05 or 0.01

The PCoA diagram shows the β-diversity values. As shown in Fig. 7A, the three groups were clearly separated, indicating that the composition of GM in ileum of MCS, MCD and DMY groups differed significantly. Figure 7B-D showed the top 10 most abundant GM at the phylum, family and level genus, respectively. At the phylum level, *Firmicutes*, *Proteobacteria* and *Bacteroidota* are the three predominant phyla. Compared with MCS group, the content of *Firmicutes* increased, while that of *Verrucomicrobiota* and *Actinobacteria* reduced in MCD group. DMY treatment significantly reduced the ratio of F/B, and elevated the contents of *Verrucomicrobiota* and *Actinobacteria* (Fig. 7E-F). At the family level, *Erysipelotrichaceae*, *Enterobacteriaceae* and *Akkermansiaceae* are the main family. MCD mice showed lower contents of *Bifidobacteriaceae* and *Akkermansiaceae* than those of MCS group mice. DMY treatment increased the contents of *Akkermansiaceae* and reduced the contents of *Erysipelotrichaceae* as compared to MCD group (Fig. 7G). *Faecalibacuium*, *Klebsiella* and *Akkermansia* are the main genera of genus level. There was no statistical difference of contents of *Faecalibacuium* and *Akkermansia* between MCS group and MCD group, while DMY treatment significantly reduced the contents of *Faecalibaculum* and increased the contents of *Akkermansia* in MCD mice (Fig. 7H).

### Effects of DMY on serum BAs profile

**Fig. 8:**
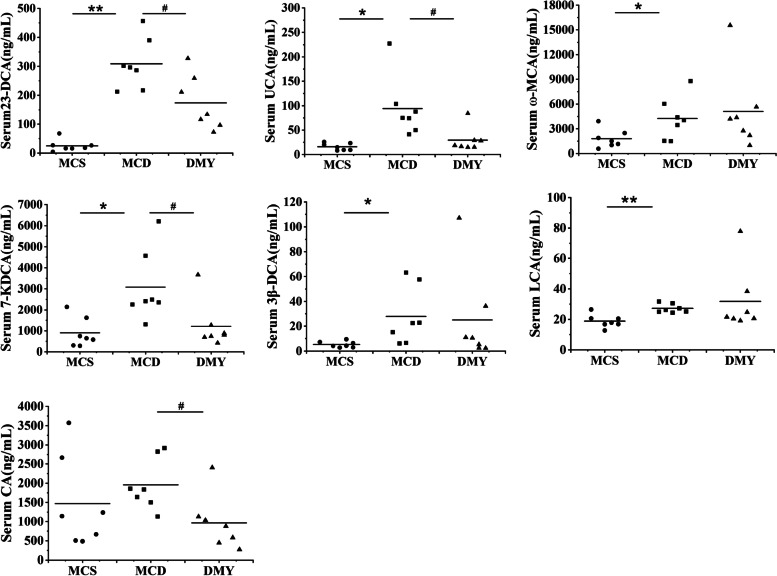
The content of differential serum BAs of the tested mice (*n* = 7). * or ** indicates comparison with MCS group and *P* < 0.05 or 0.01. # indicates comparison with MCD group and *P* < 0.05

The 50 serum BAs contents were displayed in Table S2. Compared to MCS group, the contents of 23-nor-deoxycholic acid (23-norDCA), ursocholic acid (UCA), 7-ketodeoxycholic acid (7-KDCA), ω-muricholic acid (ω-MCA), 3β-DCA and lithocholic acid (LCA) in MCD group were significantly increased. After 8 weeks of treatment with DMY, the levels of 23-DCA, UCA, 7-KDCA and cholic acid (CA) were reduced significantly (Fig. 8).

### Effect of DMY on mRNA expression of bile acid homeostasis related genes

**Fig. 9:**
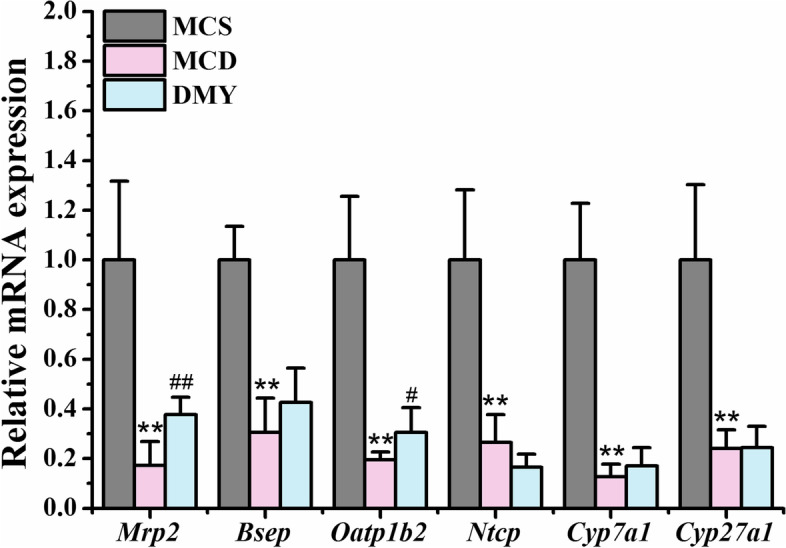
The liver mRNA expression of BA homeostasis related genes in tested mice (*n* = 6). ** indicates comparison with MCS group and *P* < 0.01. # or ## indicates comparison with MCD group and *P* < 0.05 or 0.01

Next, we studied the effect of DMY on mRNA expression of BA homeostasis related genes in liver. As presented in Fig. 9, Compared to MCS group, the mRNA expression levels of *Cyp7a1* (cholesterol 7α-hydroxylase), *Ntcp* (sodium taurocholate co-transporting polypeptide), *Bsep* (bile salt export pump), *Mrp* (multidrug resistance protein) 2, *Oatp1b2* (organic anion transporting polypeptide) and *Cyp27a1* (sterol 27-hydroxylase) in MCD group were significantly reduced. DMY treatment remarkably increased the mRNA expression of *Mrp2* and *Oatp1b2.* However, the expression of other genes did not change significantly after DMY treatment.

### Correlation between differential GM and differential lipids or BAs induced by DMY

**Fig. 10:**
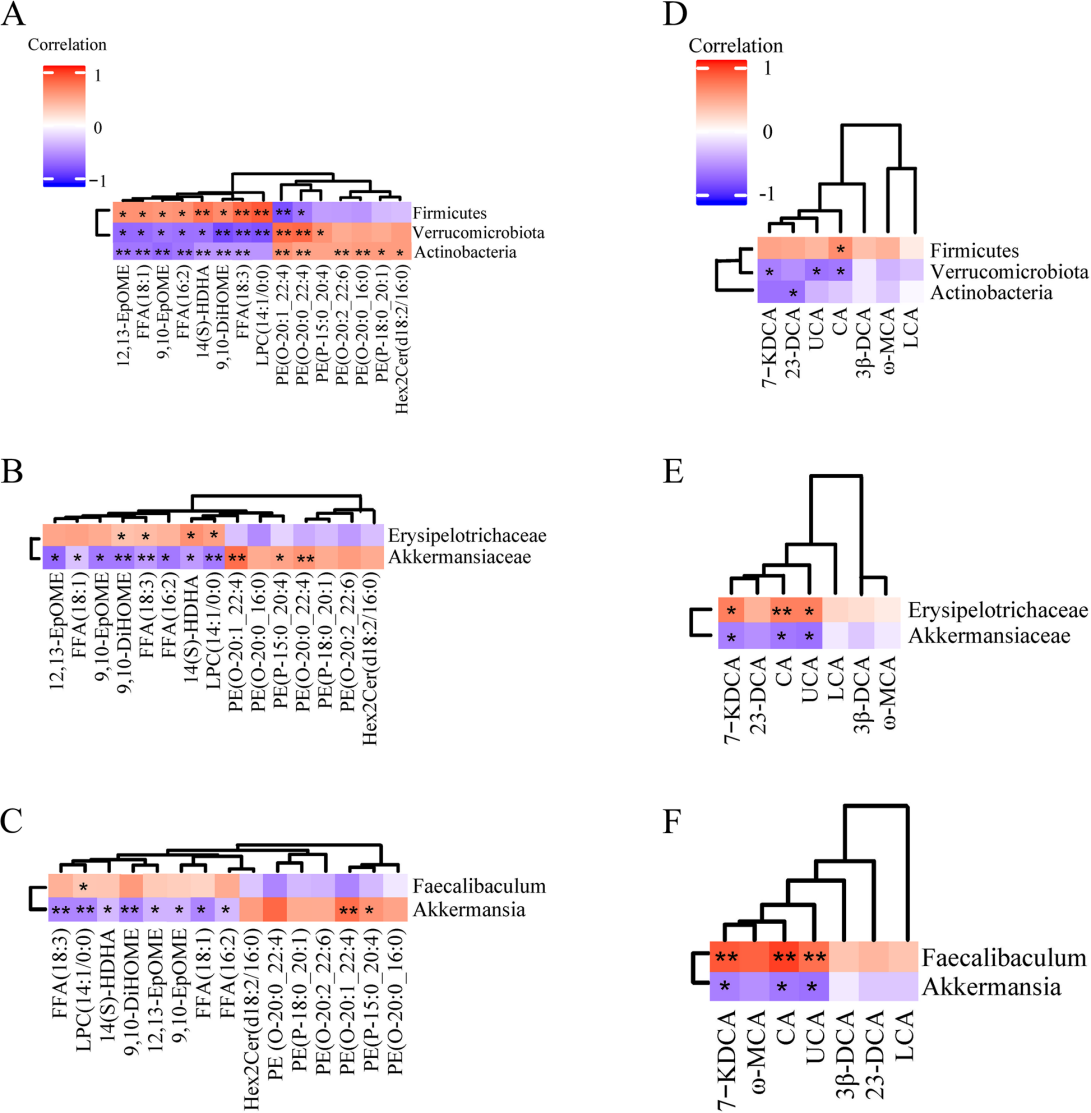
The correlations between GM at phylum level (**A**, **D**), family level (**B**, **E**) or genus level (**C**, **F**) and serum lipids or BAs in DMY-treated MCD mice, respectively. Correlation coefficient significance test: * indicates *P* < 0.05 and ** indicates *P* < 0.01

At the phylum level, *Firmicutes* was positively related with 12,13-EpOME, 14(S)-HDHA, 9,10-DiHOME, 9,10-EpOME, FFA(18:1), LPC14:1/0:0, FFA(16:2), FFA(18:3) and negatively correlated with PE(O-20:0_22:4) and PE(O-20:1_22:4). *Verrucomicrobiota* was positively related with PE(P-15:0_20:4), PE(O-20:0_22:4), and PE(O-20:1_22:4), negatively correlated with 12,13-EpOME, 14(S)-HDHA, 9,10-EpOME, 9,10-DiHOME, FFA(18:1), FFA(16:2), LPC(14:1/0:0) and FFA(18:3). *Actinobacteria* was positively related with PE(O-20:2_22:6), PE(O-20:0_16:0), PE(P-18:0_20:1), PE(O-20:0_22:4), PE(O-20:1_22:4), negatively correlated with 14(S)-HDHA, 9,10-EpOME, FFA(18:1), 9,10-DiHOME, FFA(16:2), 12,13-EpOME, FFA(18:3) (Fig. 10A). At the family level, *Erysipelotrichaceae* was positively related with FFA(18:3), 9,10-DiHOME, LPC(14:1/0:0) and 14(S)-HDHA. *Akkermansiaceae* was negatively correlated with 14(S)-HDHA, 12,13-EpOME, FFA(18:1), 9,10-EpOME, FFA(16:2), 9,10-DiHOME, FFA(18:3) and LPC(14:1/0:0), positively related with PE(O-20:0_22:4), PE(P-15:0_20:4) and PE(O-20:1_22:4) (Fig. 10B). At the genus level, *Faecalibaculum* was positively related with LPC(14:1/0:0), *Akkermansia* was negatively correlated with FFA(18:3), 9,10-DiHOME, FFA(18:1), 14(S)-HDHA, 12,13-EpOME, FFA(16:2), 9,10-EpOME and LPC(14:1/0:0) positively related with PE(O-20:1_22:4) and PE(P-15:0_20:4) (Fig. 10C).

The correlation between the differential GM and the differential serum BAs induced by DMY treatment was shown in Fig. 10D-E. *Actinobacteria* and *Firmicutes* were positively related with CA, *Verrucomicrobiota* was negatively correlated with 7-KDCA, UCA and CA at the phylum level (Fig. 10D). At the family level, *Bifidobacteriaceae* was negatively correlated with 23-DCA, *Erysipelotrichaceae* was positively related with 7-KDCA, UCA and CA, *Akkermansiaceae* was negatively correlated with 7-KDCA, UCA and CA (Fig. 10E). At the genus level, *Faecalibaculum* was positively related with 7-KDCA, UCA and CA, while *Akkermansia* was just opposite to that of *Faecalibaculum* (Fig. 10F).

## Discussion

NASH is closely associated with lipid metabolism disorders [26]. The accumulation of liver lipids can be attributed to the amount of fatty acids obtained by the liver exceeding its processing capacity, which is one of the pathogenesis of NASH [27]. Abnormal increase of FFAs level in blood will directly stimulate FFAs uptake in liver, which stimulated TNF-α expression and lead to accumulation of TG and liver steatosis [28, 29]. Oxidative lipids generated by the oxidation of polyunsaturated fat acids, such as 12-HETE, leukotriene B4 and leukotriene D4, have been shown to be associated with obesity, type 2 diabetes and insulin resistance (IR) [30–32]. PE is a precursor to synthesize phosphatidylcholine (PC), which is involved the synthesis of VLDL. Intrahepatic TG is mainly transported out of the liver in the form of VLDL. If the synthesis of PE is inhibited, the synthesis of PC is reduced, which leads to reduced TG output in the liver. DMY treatment reduced the levels of 3 FFAs (18:1, 16:2 and 18:3) and 4 oxidative lipids [9,10-EpOME, 14(S)-HDHA) 9,10-DiHOME and 12,13-EpOME], and increased the levels of 6 PEs(P-18:0_20:1, O-20:0_22:4, P-15:0_20:4, O-20:2_22:6, O-20:1_22:4 and O-20:0_16:0) in serum of MCD mice, suggesting that the improvement of DMY on MCD mice is closely related to its regulation of the serum FFAs, oxidative lipids and PEs stated above.

GM participates in the occurrence and development of NASH through mediating energy metabolism and IR [17, 33]. Dysregulation of GM can release a large amount of lipopolysaccharide and activate liver inflammation by damaging mucosal barrier [34]. Transplanting GM from HFD mice into the intestines of normal mice significantly increased IR and body fat content [35]. HFD up-regulated the gut ratio of F/B in obese mice, which promoted the body to obtain energy and lead to obesity [36]. DMY has been reported to significantly alter the richness and diversity of GM in some animal models [37], alleviated intestinal dysbiosis in colitis mice by increasing the contents of *Akkermansia* and *Lactobacillaceae* [38], improved the contents of *Bacterioidetes* and suppressed *Firmicutes* in the intestinal tract of HFD mice [39]. Similarly, the present work found that DMY treatment significantly increased the contents of *Actinobacteria*, *Verrucomicrobiota* and *Akkermansiaceae*, decreased the ratio of F/B and contents of *Erysipelotrichaceae* and *Faecalibaculum* in MCD mice. Studies have found that *Actinobacteria* can promote energy metabolism and reduce fat content [40, 41]. *Verrucomicrobia* is mainly distributed in the intestinal mucus layer, *Akkermansia* is its dominant bacterium [42]. The abundance of *Akkermansia* is positively related with body health status, such as relieving obesity and IR [43], preventing fatty liver and maintaining intestinal homeostasis by regulatin*g* liver lipid synthesis and inflammation [44]. *Faecalibactaculum* belongs to *Erysipelotrichia* in *Firmicutes* [45]*. Erysipelotrichia* is an important bacterial marker for the susceptibility to fatty liver disease caused by choline deficiency [46]. Thereby, the alleviation of DMY on MCD mice may be closely related to its inhibition of harmful bacteria and induction of beneficial bacteria in intestinal tract.

It is well known that BAs, as signaling molecules, can regulate their self-synthesis, glucolipid metabolism, GM composition and energy homeostasis through various receptors. The contents of serum GCA, TCA and GCDCA in NASH patients were higher than those in health individuals [47], and the liver damage of NASH patients was related to abnormal changes of serum BAs, for instance, increased GCA and CA in plasma were positively connected with liver inflammation [48]. CA is a hydrophobic BA that can damage mitochondrial electron transport chain, and lead to ROS and oxidative stress [49]. Increased CA level was associated with hepatocyte ballooning in NAFLD patients [50], and with increased ratio of *Firmicutes* to *Bacteroides* in rats and mice [51, 52]. Additionally, the elevated level of some secondary BAs (free and conjugated UDCA, 7-KDCA, etc.) in gut promoted the synthesis and excretion of BAs with irritable bowel syndrome patientsby inhibiting intestinal FXR/ FGF19 signaling pathway [44, 53], which was beneficial to maintain in vivo glucose homeostasis [54]. DCA is a highly toxic secondary BA that produces cytotoxicity through activation of JNK1 pathway [55], and is associated with ballooning of hepatocytes [48]. DCA is also a natural hepatic FXR antagonist. High concentration of DCA not only inhibited hepatic glycogen synthesis and promoted gluconeogenesis [10], but also inhibited the growth and reproduction of *Bacteroidete* that can improve IR [56]. The results indicated that LCA, 23-DCA, UCA, 7-KDCA, ω-MCA, 7-KDCA and 3β-DCA in MCD mice were significantly increased as compared to MCS mice, while DMY treatment reduced the contents of 23-DCA, UCA, 7-KDCA and CA. To know the potential signaling mechanism of DMY alters serum BAs content, the effect of DMY on the mRNA expression of BAs homeostasis related genes in liver of MCD mice was also detected. Primary BAs are synthesized though both classical and alternative pathways of cholesterol metabolism. Cyp7a1 and Cyp27a1 are main rate-limiting enzymes of classical and alternative pathways, respectively [13]. After binding with taurine (rats and mice) or glycine (human), the primary BAs are transported through Bsep and Mrp2 to form micelles with substances such as cholesterol and phospholipids, and stored in the gallbladder in the form of bile [7]. As well known, 95% of BAs is reabsorbed into the portal vein at the end of the ileum and circulated to the liver, then absorbed into hepatocytes by Ntcp and Oatp1b2 [7]. The results indicated that the mRNA expression levels of hepatic *Cyp7a1*, *Cyp27a1*, *Bsep*, *Mrp2*, *Ntcp* and *Oatp1b2* in MCD mice were significantly decreased as compared to MCS mice, which was basically consistent with reported literatures [57, 58]. DMY treatment significantly increased the mRNA expression levels of *Mrp2* and *Oatp1b2* in MCD mice. Up-regulated *Mrp2* can reduce the accumulation of hepatic BAs, and up-regulated *Oatp1b2* can promote BAs uptake by hepatocytes from blood. Song et al.found that DMY alleviated obesity by up-regulating the genes related to BA conjugation (*Bacs* and *Bat*) and secretion (*Bsep*, *Mrp2*, *Abcg5* and *Abcg8*), and down-regulating the genes related to bile acid re-absorption (*Asbt*, *Ostα* and *Ostβ*) of liver in obese *ob/ob* mice [21]. The increased hepatic *Mrp2* and *Oatp1b2* and decreased serum 23-DCA, UCA, 7-KDCA and CA observed in MCD mice indicated that DMY regulated BAs homeostasis of MCD mice partly due to its effect on hepatic BAs transporter expression, partly due to its effect on the abundance of certain GM.

Indeed, GM is closely related with the metabolism of lipids and BAs. Primary BAs secreted into gut are treated by GM-expressed bile salt hydrolysase and 7α/β dehydroxylase to generate secondary BAs [59]. BAs affect FXR signaling and TGR5 signal transduction in liver and intestine through enterohepatic circulation, and then regulate BA synthesis, lipid metabolism and inflammation [58, 60]. Additionally, BAs in gut can directly affect the composition of GM due to its antibacterial properties [61], or as a signal molecule to affect the expression of genes encoding antimicrobial peptides and lectins by activating BA receptors such as FXR [62], indirectly affecting GM [63]. It's worth noting that DMY induced regulatory trends on beneficial bacteria in ileum and beneficial lipids or BAs in serum of MCD mice were basically the same. More specifically, DMY treatment up-regulated probiotics (*Akkermansia* and *Bifidobacteriaceae*) and beneficial lipids [PE(O-20:0:4), PE (O-20:1:22.4), PE(P-15:0:4)], decreased harmful lipids [12, 13-EPOME, 9, 10-DIHOME, FFA(18:1), 14(S)-HDHA, FFA(16:2), LPC(14:1/0:0), FFA(18:3)] and harmful BAs (23-DCA and CA), and so it is for the regulatory trends of harmful bacteria (*Erysipelotrichaceae* and *Faecalibaculum*) (Fig. 10).

### Strengths and limitations

In the study, serum lipids and BAs in mice were detected for the first time, and the 15 lipids may be biomarkers for DMY to exert a therapeutic effect. Moreover, the correlation between the changed differential lipid metabolites and BAs and gut microbiota was analyzed. The results suggest that DMY may affect lipid metabolism and BAs by regulating GM in NASH mice induced by MCD diet.

At the same time, our study had some limitations. We found that DMY may influence BA metabolism through GM, but we did not detect fecal BAs and the expression levels of BAs metabolite-related genes in intestinal tissues. In the future work, we will conduct a more complete and in-depth study.

## Conclusions

In summary, DMY treatment increased the levels of probiotics (*Actinobacteria*, *Verrucomicrobiota*, *Bacteroidota* and *Akkermansia*) in ileum and beneficial lipids (PE O-20:1:22.4, PE O-20:0:4 and PE P-15:0:4) in serum, decreased the levels of harmful bacteria (*Erysipelotrichaceae* and *Faecalibaculum*) and harmful lipids (12, 13-EPOME, 14(S)-HDHA, 9, 10-DIHOME and LPC 14:1/0:0, etc.) and harmful BAs (23-DCA and CA). These changes should be conducive to the ameliorative effect of DMY on MCD mice. DMY can be used as a functional food for for NASH prophylaxis in daily life and clinical practice.

## Supplementary Information

**Additional file 1:** **Table S1.** Primer sequences used for RT-qPCR of the tested genes and RT-qPCR reaction conditions. **Table S2.** The serum bile acids levels (*n*=7).

## Data Availability

All data related to this study is available upon request.
