# Supplementary material for: Dihydromyricetin ameliorated nonalcoholic steatohepatitis in mice by regulating the composition of serous lipids, bile acids and ileal microflora
Source: Lipids Health Dis. 2023 Aug 2;22:112. doi: 10.1186/s12944-023-01871-7 (PMC10394885; doi:10.1186/s12944-023-01871-7)
Supplement: Supplementary file 1 — Additional file 1: Table S1. Primer sequences used for RT-qPCR of the tested genes and RT-qPCR reaction conditions. Table S2. The serum bile acids levels (n=7). [file 12944_2023_1871_MOESM1_ESM.docx]

**Table S1.** Primer sequences used for RT-qPCR of the tested genes and RT-qPCR reaction conditions.

| Name | Accession number |  | Sequences |
| --- | --- | --- | --- |
| *β-actin* | NM_007393 | Forward  Reverse | 5´-AGAGGGAAATCGTGCGTGAC-3´  5´-CAATAGTGATGACCTGGCCGT-3´ |
| *CYP7A1* | NM_007824 | Forward  Reverse | 5´-GGGAATGCCATTTACTTGGA-3´  5´-GTCCGGATATTCAAGGATGC-3´ |
| *CYP27A1* | NM_024264 | Forward  Reverse | 5´-CTATGTGCTGCACTTGCCC-3´  5´-GGGCACTAGCCAGATTCACA-3´ |
| *Bsep* | NM_021022 | Forward  Reverse | 5´-CCAGAACATGACAAACGGAA-3´  5´-AAGGACAGCCACACCAACTC-3´ |
| *Mrp2* | NM_013806 | Forward  Reverse | 5´-TCCAGGACCAAGAGATTTGC-3´  5´-TCTGTGAGTGCAAGAGACAGGT-3´ |
| *Ntcp* | NM_011387 | Forward  Reverse | 5´-AGGGGGACATGAACCTCAG-3´  5´-TCCGTCGTAGATTCCTTTGC-3´ |
| *Oatp1b2* | NM_020495 | Forward  Reverse | 5´-ACCAAACTCAGCATCCAAGC-3´  5´-TAGCTGAATGAGAGGGCTGC-3´ |
| reaction conditions | 95°C for 5 min, 95°C for 30 s, 56.3°C for 30 s, 72°C for 30 s, performed for 40 cycles | | |

**Table S2.** The serum bile acids levels (n=7).

| Class | Class | MCS group  (ng/mL) | MCD group  (ng/mL) | DMY group  (ng/mL) |
| --- | --- | --- | --- | --- |
| taurolithocholic acid-3-sulfate | TLCA-3S | / | / | / |
| Dehydrolithocholic acid | DLCA | / | / | / |
| Isoallolithocholic acid | IALCA | / | / | / |
| isolithocholic acid | ILCA | / | / | / |
| Lithocholic acid | LCA | 18.84±3.95 | 27.18±2.65 | 31.78±19.78 |
| Nor-Deoxycholic Acid | 23NorDCA | 25.20±17.89 | 308.88±49.47 | 173.90±60.01 |
| 3-oxodeoxycholic acid | 3-oxo-DCA | 19.10±13.33 | 41.04±26.12 | 55.74±83.93 |
| 7-ketolithocholic acid | 7-KLCA | 9.45±7.36 | 18.11±11.74 | / |
| 12-ketolithocholic acid | 12-KLCA | / | / | 16.30±12.28 |
| Deoxycholic acid | DCA | 302.09±167.80 | 541.05±274.35 | 448.77±369.08 |
| Isodeoxycholic acid | IDCA | / | / | / |
| murideoxycholic acid | MDCA | 31.44±32.79 | 17.31±13.02 | 24.42±37.11 |
| 3β-deoxycholic acid | 3β-DCA | 5.31±2.28 | 27.75±21.65 | 24.99±35.23 |
| 3β-Ursodeoxycholic Acid | 3β-UDCA | 4.79±3.59 | 6.88±2.72 | 7.92±6.57 |
| Chenodeoxycholic acid | CDCA | 265.08±159.30 | 516.86±354.88 | 298.76±430.08 |
| β-Hyodeoxycholic Acid | 3β-HDCA | / | / | / |
| Ursodeoxycholic acid | UDCA | 15.95±6.74 | 94.32±57.77 | 29.53±23.05 |
| Hyodeoxycholic acid | HDCA | 57.19±54.09 | 44.95±11.31 | 34.95±28.60 |
| norcholic acid | NCA | / | 38.59±11.48 | 32.14±9.04 |
| Dehydrocholic acid | DHCA | / | 2.73±0.78 | 2.32±1.09 |
| 6,7-diketolithocholic acid | 6,7-DKLCA | / | / | / |
| 7,12-diketolithocholic acid | 7,12-DKLCA | / | 10.36±3.95 | / |
| 12-Oxochenodeoxycholic acid | 12-oxo-CDCA | / | / | / |
| 3-Oxocholic acid | 3-oxo-CA | 3.95±3.64 | 5.50±2.92 | 2.75±3.44 |
| 7-Ketodeoxycholic acid | 7-KDCA | 902.78±652.38 | 3084.70±1563.15 | 1208.76±1028.19 |
| ω-muricholic acid | ω-MCA | 1784.80±1037.18 | 4240.15±2372.45 | 5089.00±4502.66 |
| 3β-Cholic Acid | 3β-CA | / | / | / |
| Cholic acid | CA | 1468.42±1104.71 | 1959.49±620.92 | 962.50±655.98 |
| β-muricholic acid | β-MCA | 828.34±860.40 | 2399.63±1651.48 | 2445.53±3194.77 |
| hyocholic acid | HCA | 15.04±10.14 | 65.27±52.28 | 27.89±29.38 |
| α-muricholic acid | α-MCA | 110.75±110.73 | 217.14±118.22 | 132.26±140.30 |
| Ursocholic acid | UCA | 15.04±5.66 | 111.91±54.98 | 130.84±122.08 |
| Glycolithocholic acid | GLCA | 1.66±0.19 | 1.82±0.30 | 1.23±0.13 |
| Glycodeoxycholic acid | GDCA | / | 0.34±0.17 | 0.27±0.11 |
| Glycoursodeoxycholic acid | GUDCA | / | / | / |
| Glycochenodeoxycholic acid | GCDCA | 0.31±0.16 | 0.33±0.06 | 0.26±0.03 |
| lithocholic acid-3-sulfate | LCA-3S | / | / | / |
| Glycodehydrocholic acid | GDHCA | / | / | / |
| Glycocholic acid | GCA | / | 10.52±2.18 | 10.83±1.66 |
| Glycohyocholic acid | GHCA | / | / | / |
| taurolithocholic acid | TLCA | 28.35±31.33 | 15.93±2.89 | 12.95±2.56 |
| Taurochenodeoxycholic acid | TCDCA | 715.62±748.51 | 400.37±122.184 | 440.00±99.77 |
| Taurodeoxycholic acid | TDCA | 381.67±352.97 | 244.78±77.53 | 189.24±89.94 |
| Tauroursodeoxycholic acid | TUDCA | 427.43±368.43 | 266.75±81.38 | 256.01±65.83 |
| Taurodehydrocholic acid | TDHCA | / | / | / |
| glycolithocholic acid-3-sulfate | GLCA-3S | / | / | / |
| Taurohyocholic acid | THCA | / | / | / |
| Tauro-β-muricholic acid | Tβ-MCA | 9614.02±11017.94 | 7787.08±2299.32 | 7517.05±1307.35 |
| Taurocholic acid | TCA | 11325.03±12396.73 | 5186.67±1261.64 | 6133.73±1041.95 |
